# A large-scale dataset reveals taxonomic and functional specificities of wild bee communities in urban habitats of Western Europe

**DOI:** 10.1038/s41598-022-21512-w

**Published:** 2022-11-07

**Authors:** Arthur Fauviau, Mathilde Baude, Nicolas Bazin, William Fiordaliso, Alessandro Fisogni, Laura Fortel, Joseph Garrigue, Benoît Geslin, Jérémie Goulnik, Laurent Guilbaud, Nina Hautekèete, Charlène Heiniger, Michael Kuhlmann, Olivier Lambert, Dominique Langlois, Violette Le Féon, Carlos Lopez Vaamonde, Grégory Maillet, François Massol, Nadia Michel, Alice Michelot-Antalik, Denis Michez, Hugues Mouret, Yves Piquot, Simon G. Potts, Stuart Roberts, Lise Ropars, Lucie Schurr, Colin Van Reeth, Irène Villalta, Vincent Zaninotto, Isabelle Dajoz, Mickaël Henry

**Affiliations:** 1 Sorbonne Université, UPEC, Université Paris Cité, CNRS, IRD, INRAE, iEES-Paris, Paris, France; 2 Université d’Orléans, Orléans, France; 3 Sorbonne Université, UPEC, Université Paris Cité, CNRS, IRD, INRAE, Institut d’Ecologie et des Sciences de l’Environnement (iEES-Paris), Paris, France; 4 RNN des Gorges de l’Ardèche, Saint-Remèze, France; 5 Université de Mons, Mons, Belgium; 6 UMR 8198, Evo-Eco-Paleo, CNRS, Université de Lille, Lille, France; 7 INRAE, Unité de Recherche Abeilles & Environnement, Avignon, France; 8 RNN Massane, Argelès-sur-Mer, France; 9 Aix Marseille Université, Université d’Avignon, CNRS, IRD, IMBE, Campus Étoile, Faculté des Sciences St-Jérôme, Marseille, France; 10 LAE, Université de Lorraine, INRAE, Nancy, France; 11 Association Noé, Paris, France; 12 HEPIA, University of Applied Sciences and Arts Western Switzerland, Geneva, Switzerland; 13 Zoological Museum, University of Kiel, Kiel, Germany; 14 Centre Vétérinaire de la Faune Sauvage et des Ecosystèmes/Oniris, Nantes, France; 15 Conservatoire d’espaces Naturels de Franche-Comté, Cléron, France; 16 UR633, Zoologie Forestière, INRAE, Orléans, France; 17 IRBI, UMR7261 CNRS, Université de Tours, Tours, France; 18 RNN Tourbière du Grand Lemps, Châbons, France; 19 U1019 - UMR 9017 - CIIL - Center for Infection and Immunity of Lille, Université de Lille, CNRS, Inserm, CHU Lille, Institut Pasteur de Lille, Lille, France; 20 ARTHROPOLOGIA, La-Tour-De-Salvagny, France; 21 University of Reading, Berkshire, Great Britain; 22 ThéMA, UMR 6049 CNRS, Université Bourgogne-Franche-Comté, Besançon, France; 23 Biogéosciences, UMR 6282 CNRS, Université Bourgogne-Franche-Comté, Dijon, France; 24 Research Center for Alpine Ecosystems, Chamonix-Mont-Blanc, France; 25 Université Paris Cité, Paris, France

**Keywords:** Ecology, Biodiversity, Community ecology, Urban ecology

## Abstract

Wild bees are declining, mainly due to the expansion of urban habitats that have led to land-use changes. Effects of urbanization on wild bee communities are still unclear, as shown by contrasting reports on their species and functional diversities in urban habitats. To address this current controversy, we built a large dataset, merging 16 surveys carried out in 3 countries of Western Europe during the past decades, and tested whether urbanization influences local wild bee taxonomic and functional community composition. These surveys encompassed a range of urbanization levels, that were quantified using two complementary metrics: the proportion of impervious surfaces and the human population density. Urban expansion, when measured as a proportion of impervious surfaces, but not as human population density, was significantly and negatively correlated with wild bee community species richness. Taxonomic dissimilarity of the bee community was independent of both urbanization metrics. However, occurrence rates of functional traits revealed significant differences between lightly and highly urbanized communities, for both urbanization metrics. With higher human population density, probabilities of occurrence of above-ground nesters, generalist and small species increased. With higher soil sealing, probabilities of occurrence of above-ground nesters, generalists and social bees increased as well. Overall, these results, based on a large European dataset, suggest that urbanization can have negative impacts on wild bee diversity. They further identify some traits favored in urban environments, showing that several wild bee species can thrive in cities.

## Introduction

Insects are declining worldwide^1,2^, and the main drivers of this decline are habitat loss and changes in land-use^3–6^. Pollinating insects are declining as well^7^, mostly due to agricultural intensification and urbanization^8,9^. Given the expected expansion of cities in the near future^10^, it appears crucial to understand the effects of urbanization on pollinator communities.

Urban areas have long been seen as inhospitable habitats for pollinators, resulting in species-poor pollinator communities. One of the reasons is tied to the high proportion of impervious surfaces, that has often been linked to a decrease in abundance and diversity of wild bees, due to, among other reasons, floral resource depletion with increased soil sealing^7,11,12^. Also, the urban heat island effect, in which cities exhibit warmer temperatures than surrounding environments, can have a negative effect on pollinators, as illustrated by a 41% decrease in the abundance of wild bees per 1 °C mean temperature increase in the city of Raleigh, USA^12^. Likewise, pollinator abundance and diversity are negatively impacted by chemical pollution in cities^13,14^. However, not all studies find negative relationships between pollinator communities and urbanization, as pointed out in a recent review, emphasizing the lack of a generalizable trend concerning this issue^15^. In this review, 24% of studies report negative effects of urbanization on pollinators, versus 37% with positive effects and 39% with no effect.

Indeed, several studies have shown that pollinator communities, and especially wild bees, are not scarce in cities. Several urban environments have actually reported hosting a substantial diversity and abundance of wild bees^16–21^. For example, 146 wild bee species have been reported in the city of Paris, France^22^, and 291 in the urban and suburban areas of Lyon (France), representing around one third of the French bee fauna^16^. In the city of Poznan (Poland), more than 100 wild bee species have been reported, representing 25% of the Polish fauna^20^. Some authors even consider cities as “hotspots” for pollinators^23^, as urban-specific habitats, such as private gardens and allotments, support substantial pollinator diversity levels^24^. Overall, this has led some authors to qualify cities as “refuges'' for pollinators, especially when compared to surrounding agricultural landscapes, due to a lower pesticide usage and more conservation efforts in cities^25^.

However, urban environments still act as an ecological filter on species assemblages^26^, meaning that species occurring in urban areas are not random samples from non-urban communities, and as such can display functional traits that are better adapted to these urban environments. Indeed, an ecological filtering pattern has been described regarding some pollinator communities^27–29^. More precisely, a recent review on wild bee traits related to urbanization suggests that cities are more likely to host below-ground, social and small wild bee species, as well as being more adapted to generalist and late-spring emerging species^30^. However, the same review emphasizes, again, a lack of generalizable trends from the literature.

This lack of generalizable trends both in terms of taxonomic or functional diversity may be explained by the fact that most studies of urban pollinator communities are carried out either in a single large city^12,17,19,31^, or along an urbanization gradient covering one or a few neighboring cities^11,16,20,27,32^. Also, a single proxy of urbanization level is usually used, namely the proportion of impervious surfaces^11,16,27^. Yet, landscapes with similar proportions of impervious surfaces may strongly differ in their ability to provide nesting and feeding resources for insect pollinators, as the proportion of impervious surfaces only provides a partial view of the complex urbanization process.

Here, we used two different metrics to describe urbanization: the proportion of impervious surfaces and the human population density. These two variables are complementary: the proportion of impervious surfaces only evaluates the level of soil sealing, while the human population density further reveals the density and complexity of human infrastructures. The latter variable might bring new insight into the assemblage rules of wild bee communities in cities and help explain the somehow contradictory results found in the literature. Indeed, areas with equally high covers of impervious surfaces might display disparate human population density values, potentially leading to different bee community diversities or functional profiles. For example, we expect that cities having a high human population density will display more buildings and man-made infrastructures, thus more concrete structures that may favor above-ground cavity-nesters.

This study is also bringing together more than 800 survey sites, where wild bee communities have been inventoried in the past fifteen years. These sites are located in various regions of France, Belgium and Switzerland, and in different habitats, ranging from highly urbanized habitats to protected natural areas. The dataset gathers a total of 580 wild bee species, with more than 65,000 individual records, and was analyzed with respect to the different sampling approaches carried out by the data providers.

Our objective is to clarify the effect of urbanization on wild bee communities, by using distinct, complementary metrics of urbanization, and by identifying possible ecological filter effects of urbanization on wild bee communities and functional trait diversities. As information is available for many wild bee species about their morphological and life cycle traits^33,34^, we only focused on wild bee communities and their responses to urbanization. Also, we focused on functional traits liable to determine bee species sensitivity to habitat alterations, namely nesting behavior, sociality, body size, and diet specialization. These traits are broadly used in other studies^30^ and are thus well-established ecological attributes readily comparable among studies and less prone to errors.

We will (i) assess the possible links between local wild bee species richness (i.e., α diversity) and urbanization metrics and (ii) evaluate the changes in wild bee taxonomic and functional composition with increasing urbanization, with the underlying idea that ecological filters might lead to a taxonomic homogenization of bee communities or favoring some functional traits over others in urban areas. Analyses of bee species turnover (i.e., β diversity) using urbanization metrics, combined with analyses of occurrence rates of wild bee functional traits, may reveal taxonomic homogenization.

## Results

### Description of the dataset

The dataset includes a total of 65,380 individual wild bees, belonging to 580 species (469 non-parasitic, and 111 parasitic), representing a fair part of French, Belgian and Swiss national wild bee diversity (949, 399 and 650 wild bee species, respectively^35^). The most abundant species in the dataset are *Lasioglossum malachurum* (5203 individuals), *Bombus pascuorum* (3655 individuals), *B. gr. terrestris* (3261 individuals, including *B. terrestris* and *B. lucorum*), and *L. morio* (3179 individuals).

Human population density within a 500 m radius around each site varied from 0 in the most natural areas, to 27,076 inhabitants per km^2^ in Lyon (France), closely followed by a site in Paris (France) with 26,408 inhabitants per km^2^ (mean = 2200.8, sd = 4419.4).

The proportion of impervious surfaces within a 500 m radius varied from 0% in the most natural areas, to 100% in the most urbanized zones (mean = 38.9%, sd = 39.2). Other habitats found within a 500 m radius included semi-natural habitats (forests, grasslands, heathlands, mean = 22.3%, sd = 32.6), agricultural areas (mean = 28.1%, sd = 33.6) and water (mean = 6.1%, sd = 12.6).

The site with the largest sample was located in the city of Geneva (Switzerland) and comprised 2299 wild bee individuals, followed by three sites from the city of Lyon (France), with 1622, 1321 and 1303 wild bee individuals.

### Variations of bee community species richness with urbanization (α diversity)

**Figure 1:**
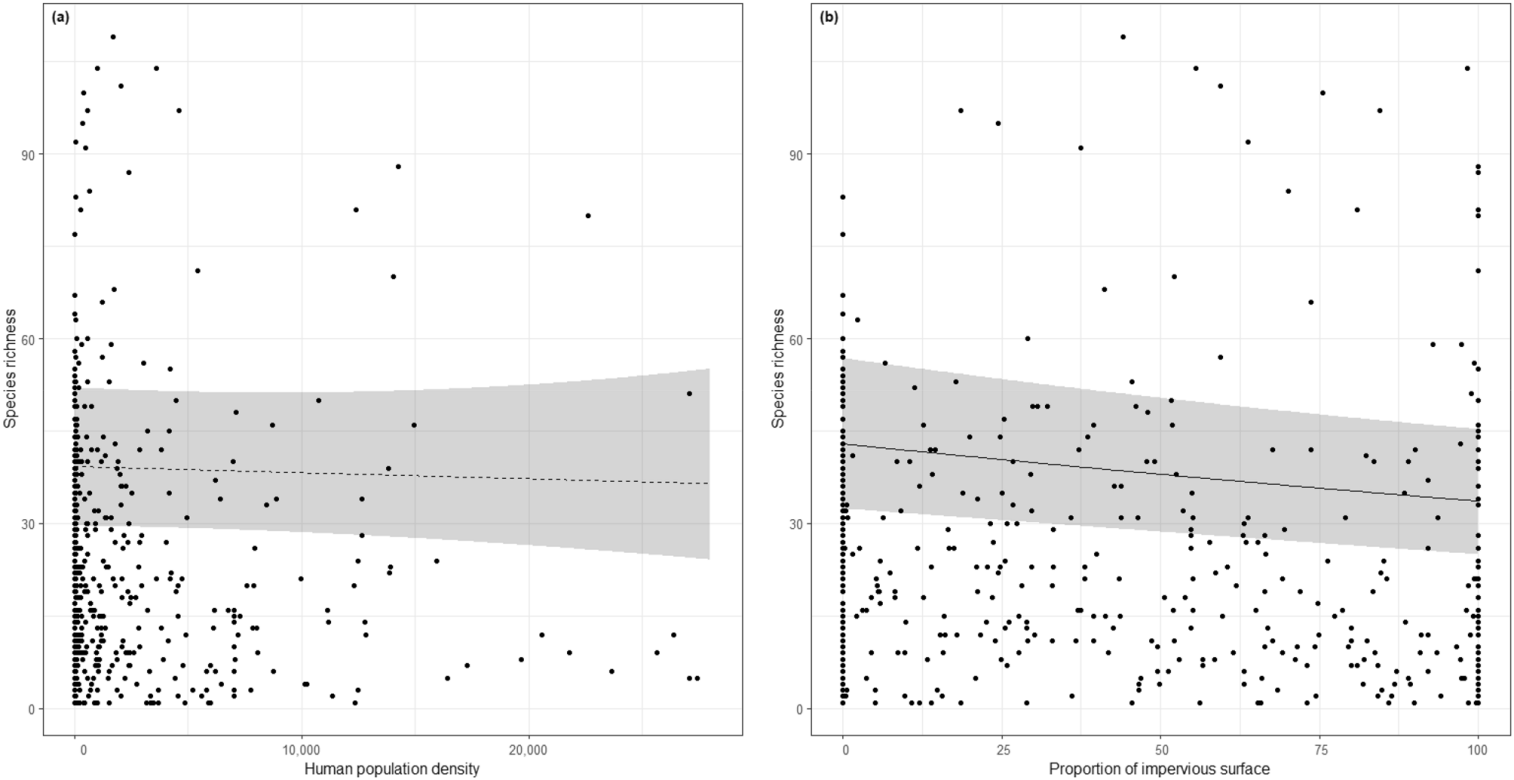
General patterns of wild bee species richness as a function of urbanization. (**a**) species richness as a function of human population density; (**b**) species richness as a function of the proportion of impervious surface. Lines represent model predictions (dashed if not significant, α = 5%), and the shaded areas stand for the 95% confidence interval of these predictions.

There was no significant effect of the human population density on community species richness (chi2 = 0.06; *p* = 0.80, Table 1, Fig. 1a). The proportion of impervious surfaces showed a significant negative relationship with the species richness of bee communities, though quite close to the significance level (coefficient = − 0.09; chi2 = 3.86; *p* = 0.0493, Table 1, Fig. 1b).

**Table 1:** Results from the species-urbanization spaMM model.

Predictor	Coefficient ± SE	df	Chi2 test	*p* value
Human population density	− 0.01 (0.04)	1	0.06	0.80
Proportion of impervious surfaces	− 0.09 (0.05)	1	3.86	**0.0493**
Biogeographical zone		3	4.31	0.23

*p* values in bold are significant (α = 5%). Coefficients for continuous variables are from the summary of the model and are given with their standard error. The *p* value and the chi square tests were evaluated using Likelihood Ratio Tests between the full model and the full model without the considered variable.

### Variations of bee community taxonomic dissimilarity with urbanization (β diversity)

**Figure 2:**
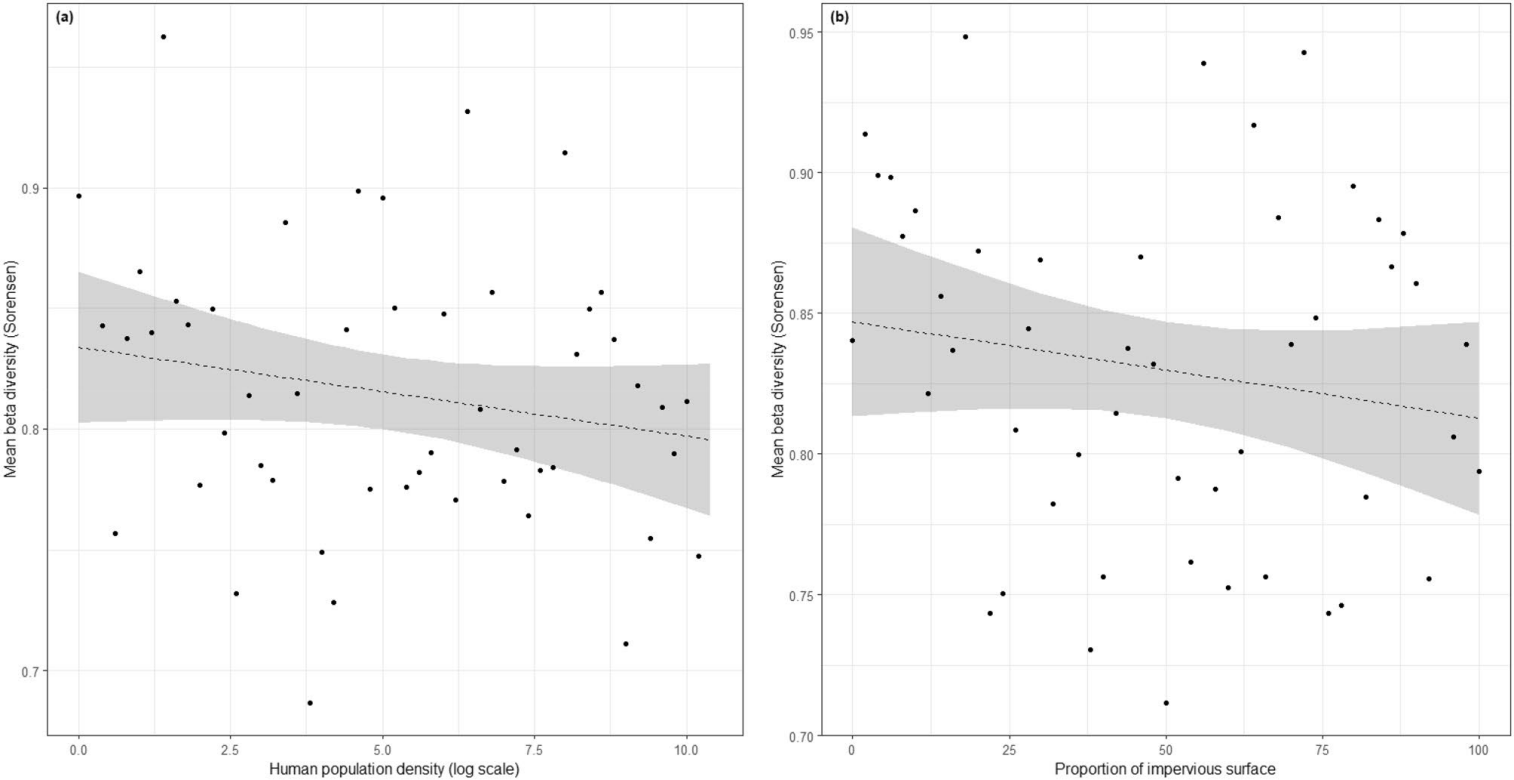
Graphs from the beta diversity model, representing (**a**) the mean pairwise beta diversity between sites having a log human population density (x-axis, log scale) of x and x + 0.2 and (**b**) the mean pairwise beta diversity between sites having a proportion of impervious surface (x-axis) of x and x + 2. The line represents model prediction (dashed if not significant, α = 5%) and the shaded areas around is the 95% confidence interval of these predictions.

There was no significant effect of the two urbanization metrics on bee community taxonomic β diversity. For human population density we found that t = − 1.38; *p* = 0.18, and for proportion of impervious surface in a 500 m radius, t = − 1.15; *p* = 0.26 (Table 2, Fig. 2).

**Table 2:** Results (summary) of the two β-diversity models.

Predictor	Coefficient (± SE)	t-value	*p* value
**Model 1**			
Human population density	− 0.004 (0.003)	− 1.38	0.18
**Model 2**			
Proportion of impervious surface	− 0.0003 (0.0003)	− 1.15	0.26

Model 1 is the β diversity depending on the human population density, and Model 2 is the β-diversity depending on the proportion of impervious surface. %). Coefficients are given with their standard error.

### Variations of community functional traits with urbanization

Here we report on how the probability of occurrence for a wild bee species depends on its traits (nesting, diet, size and sociality) and the interaction with each of the two urbanization metrics.

We found several lines of evidence that bee communities in highly urbanized areas are not a random subset of the global species pool. Indeed, species occurrence data revealed significant interactions between both urbanization metrics and several of the functional traits considered, meaning that urbanization effects differed among functional trait modalities (Tables 3 and 4).

**Table 3:** Summary of the traits-density model.

Predictor	Coefficient (± SE)	z-value	*p* value (adj)
Human population density	− 0.04 (0.05)	− 0.83	0.41
**Traits, no interaction**			
Nesting: above versus **below**	0.02 (0.18)	0. 107	0.91
Lectism: specialist versus **generalist**	− 0.54 (0.17)	− 3.16	**0.002**
Sociality: solitary versus **social**	− 0.96 (0.23)	− 4.09	**< 0.0001**
Size: large versus **small**	− 0.17 (0.16)	− 1.04	0.30
**Traits, interacting with human population density**			
Nesting: above versus **below**	0.31 (0.03)	11.43	**< 0.0001**
Lectism: specialist versus **generalist**	− 0.15 (0.03)	− 4.42	**< 0.0001**
Sociality: solitary versus **social**	− 0.05 (0.03)	− 1.68	0.09
Size: large versus **small**	− 0.14 (0.02)	− 5.76	**< 0.0001**

Predictor		Chisq	*p* value
Biogeographical zone		50.591	** < 0.0001**

*p* values in bold are significant (α = 5%). The significance of the categorical variable “Biogeographical zone” was evaluated using a Likelihood Ratio Test. This model is a mixed-effect model, the random variables included are the sites ID, nested in the sampling categories, as well as the species names. Coefficients are given with their standard error. Traits in bold are the reference modalities. *p* value(adj) means that p-values were adjusted with the False Discovery Rate adjustment method.

**Table 4:** Summary of the traits-impervious surfaces model.

Predictor	Coefficient (± SE)	z-value	*p* value (adj)
Proportion of impervious surfaces	− 0.20 (0.05)	− 3.89	**< 0.0001**
**Traits, no interaction**			
Nesting: above versus **below**	0.02 (0.18)	0.09	0.92
Lectism: specialist versus **generalist**	− 0.54 (0.17)	− 3.16	**0.002**
Sociality: solitary versus **social**	− 0.96 (0.23)	− 4.08	**< 0.0001**
Size: large versus **small**	− 0.17 (0.16)	− 1.02	0.31
**Traits, interacting with proportion of impervious surfaces**			
Nesting: above versus **below**	0.31 (0.03)	11.4	**< 0.0001**
Lectism: specialist versus **generalist**	− 0.09 (0.03)	− 3.03	**0.002**
Sociality: solitary versus **social**	− 0.08 (0.03)	− 2.68	**0.007**
Size: large versus **small**	− 0.02 (0.02)	− 0.69	0.49

Predictor		Chisq	*p* value
Biogeographical zone		56.877	** < 0.0001**

*p* values in bold are significant (α = 5%). The significance of the categorical variable “Biogeographical zone” was evaluated using a Likelihood Ratio Test. This model is a mixed-effect model, the random variables included are the sites ID, nested in the sampling categories, as well as the species names. Coefficients are given with their standard error. Traits in bold are the reference modalities. *p* value(adj) means that p-values were adjusted with the False Discovery Rate adjustment method.

**Figure 3:**
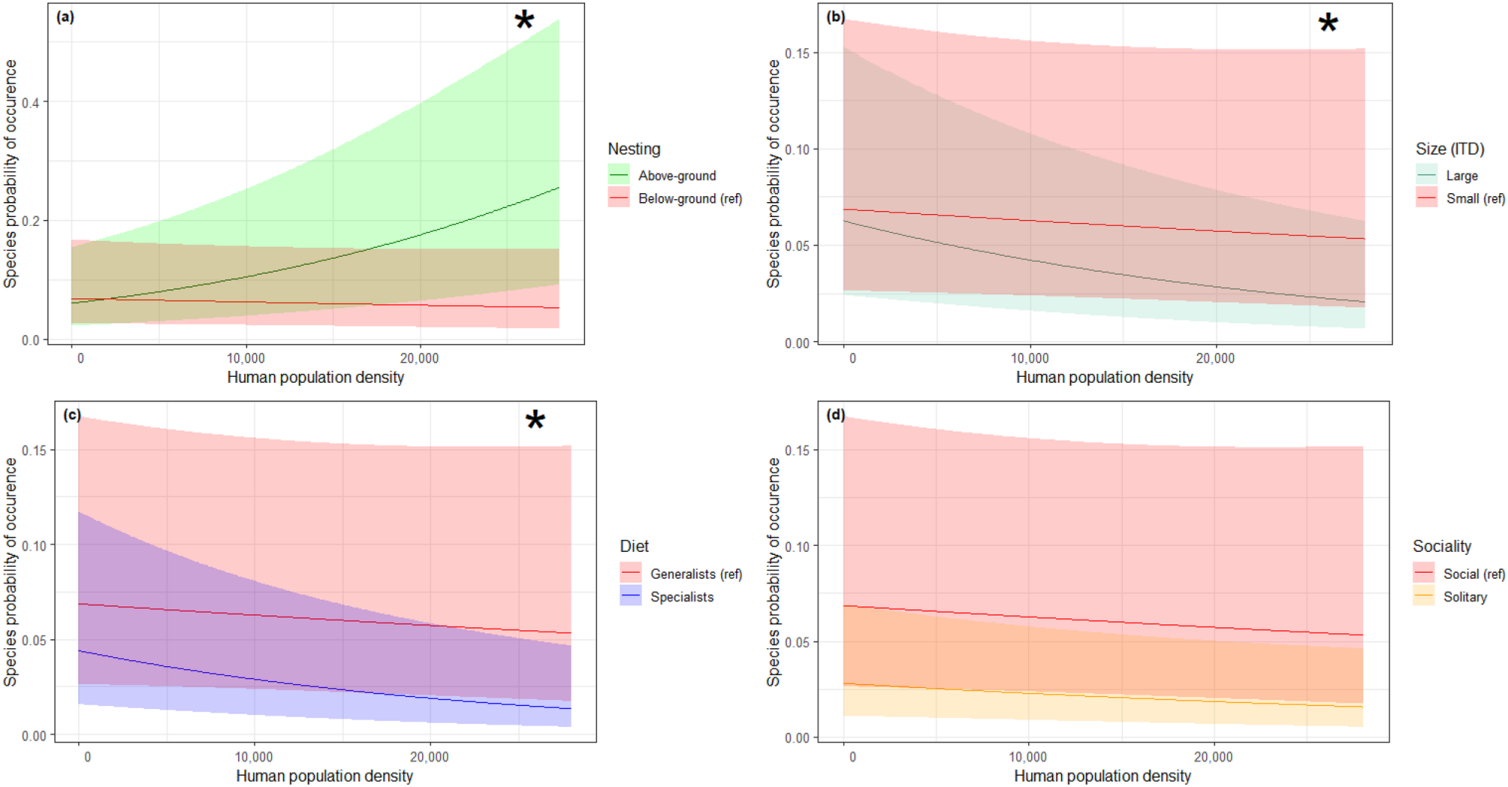
Bee species probability of occurrence as a function of human population density and functional trait modalities. Model predictions are plotted separately by trait modalities, so as to highlight the statistical interactions that may support an ecological filter hypothesis, i.e. different slopes between two modalities of a given trait. In each panel, the reference modality handled by the model (red curves, with a “ref” legend tag) was chosen on the basis of the most abundant species in the dataset, namely *Lasioglossum malachurum*, a small-sized species nesting below ground, with generalist diet and social habits. Shaded areas stand for the 95% confidence interval of these predictions. The plots labelled with an asterisk indicate a significant trait modality-human population density interaction.

Human population density exerted a significant positive effect on the occurrence probability of above-ground nesting species compared to below-ground nesting species as shown by the significant “human population density × nesting trait” interaction (z = 11.43, *p* < 0.0001). Conversely, human density was significantly more detrimental to the occurrence of specialist bee species compared to generalist ones (z = − 4.42; *p* < 0.0001), as well as for the occurrence of large bees compared to small ones (z = − 5.76; *p* < 0.0001). Overall, small-bodied, as well as generalist species were more frequently found in densely populated habitats, compared to large-bodied and specialist species (Fig. 3b,c). There was no significant difference between the occurrence patterns of social and solitary species (z = − 1.68; *p* = 0.09) (Table 3, Fig. 3).

**Figure 4:**
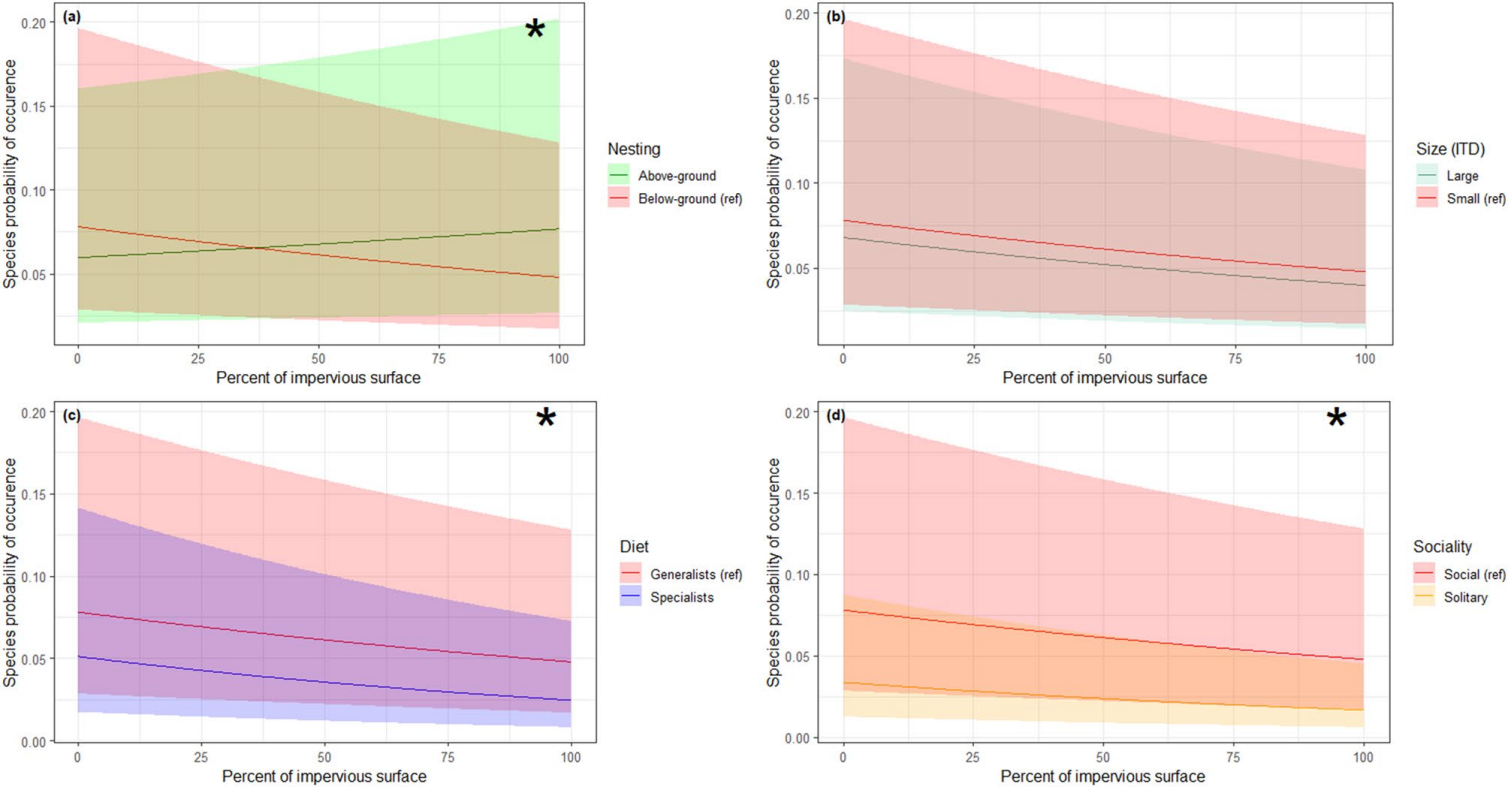
Bee species probability of occurrence as a function of the percent of impervious surface and functional trait modalities. Model predictions are plotted separately by trait modalities, so as to highlight the statistical interactions that may support an ecological filter hypothesis, i.e. different slopes between two modalities of a given trait. In each panel, the reference modality handled by the model (red curves, with a “ref” legend tag) was chosen on the basis of the most abundant species in the dataset, namely *Lasioglossum malachurum*, a small-sized species nesting below ground, with generalist diet and social habits. Shaded areas stand for the 95% confidence interval of these predictions. The plots labelled with an asterisk indicate a significant trait modality-percent of impervious surface interaction.

Similar trends were recorded concerning the responses of nesting and diet traits to the proportion of impervious surfaces (Tables 3 and 4). Again, a significantly steeper positive effect on the occurrence of above-ground nesting species compared to below-ground nesters was detected (z = 11.4; *p* < 0.0001), as well as a more negative effect of the proportion of impervious surfaces on the occurrence of specialists compared to generalists (z = − 3.03; *p* = 0.002). No significant difference between the occurrence patterns of large or small bees was detected (z = − 0.69; *p* = 0.49), but we found a significant positive effect of soil sealing on social species compared to solitary ones (z = − 2.68, *p* = 0.007, Table 4, Fig. 4).

Lastly, we found no link between human population density and wild bee species probability of occurrence (z = − 0.83; *p* = 0.41, Table 3), whereas the proportion of impervious surfaces significantly lowered wild bee species probability of occurrence (z = − 3.89; *p* < 0.0001, Table 4).

The above-ground, generalist wild bee species represented 75 (16%) of the 469 non-parasitic species, the most represented species in urban environments being from the genera *Hylaeus, Osmia* and *Anthidium,* with a majority of *H. communis, H. hyalinatus, O. bicornis, O. cornuta, A. manicatum, and A. florentinum*. *Bombus* species were found at similar rates in urban and non-urban environments, the most present in urban areas being *B. pascuorum, B. gr. terrestris and B. lapidarius* (below-ground nesters). The *Eucera* genus, a below-ground nester with the majority of species also being specialists, was mostly found in non-urban environments, with the exception of *Eucera nigrescens*, which was found in 22 urban sites.

## Discussion

Here we assessed how species and functional diversity components of wild bee assemblages responded to increasing urbanization levels, using a large dataset encompassing recent surveys gathering 838 sampling sites located in natural, semi-natural and urban habitats of France, Belgium and Switzerland.

We found a weak, but significant negative effect of the proportion of impervious surfaces in a 500 m radius around each site on local species richness of bee communities. Thus, sites with high soil sealing tended to host less species than those with low soil sealing. However, this trend was not observed when using human population density as an urbanization metric: sites with denser human populations hosted on average the same number of species as less densely populated sites.

Concerning taxonomic homogenization of communities, we did not record any effects of urbanization, both in terms of impervious surfaces or human population density.

Analyses of occurrence rates of bee functional traits revealed significant differences between poorly and highly urbanized communities, for both urbanization metrics. With higher human population density, probabilities of occurrence of above-ground nesters, generalist and small species increased, and a higher probability of occurrence of above-ground nesters, generalists and social bees were recorded in areas with high soil sealing.

Therefore, we found overall consistent results linking urbanization and wild bees taxonomic as well as functional trait diversity, even though analyses stemmed from a combination of many independent studies covering a broad range of anthropized and natural aeras from western Europe. This further highlights the greater generalizability of those ecological trends throughout European temperate biomes compared to other studies typically focusing on a single city and its immediate vicinity.

### Two complementary metrics of urbanization intensity

To quantify urbanization, we used two variables: soil sealing^12,16,19,36^ in a 500 m radius, and the mean human population density, also in a 500 m radius, the latter variable being used only recently to assess pollinator responses to urban environments^37,38^. These two variables return different but complementary information concerning urban environments. Indeed, if soil sealing gives an idea as to how human activities impact land use, human population density helps distinguish between very dense urban areas and very impervious areas with lower densities of buildings. High human population density areas are usually associated with high levels of soil sealing, but the contrary is not true. Similarly, areas with low soil sealing are usually associated with low human population densities, but again, the opposite is not always true. Therefore, we found it informative to consider both variables when analyzing the response of wild bee assemblages to urbanization.

Note that some specific habitat types, for example business districts, are exceptions to the rule. These places are indeed very densely urbanized, but with very low population density. However, no inventories have been carried out in these places, and thus will not be a problem for our study.

### Response of bee community species richness to urbanization

One of our goals was to position this study in the context of the contrasting findings on pollinator communities and urbanization. Whereas no consistent trend is reported in literature^15^, our large dataset reveals that high soil sealing is detrimental to wild bee species richness. This offers a unified view of a trend that has been unequally evidenced from studies focusing on a single or few cities only. High proportions of soil sealing reduce the availability of nesting sites for ground-nesting bee species. This may in turn lower the species diversity of local assemblages, by filtering out ground-nesting bees, leaving mainly cavity-nesting bees. Furthermore, high levels of soil sealing can lead to depletion of floral resources, of extreme importance for bees, especially in highly disturbed environments such as cities^39,40^. Note that several previous studies report the opposite, with high local species richness of wild bees in urbanized habitats. However, these positive effects are often associated with intermediate levels of urbanization^15,16^, where private gardens and other green spaces may supply abundant floral resources, in conjunction with intermediate levels of soil sealing^16–20,24^.

On the contrary, there was no significant relationship between local species richness and human population density. Recently, two recent studies have used this metric to analyze how urbanization impacts local diversity of bee, hoverfly^37^ or butterfly^38^ assemblages, and both studies report negative impacts of human population density. However, high levels of human population density do not necessarily correlate with low availability of floral resources or nesting sites for pollinating insects. Several studies show that densely-populated urban environments may be adequate habitats for pollinating insects, due to alternative management practices of urban green space^41^ and the year-round availability of ornamental flowers^42,43^. Here, the absence of a clear effect of human population density on local bee species richness masks a change in the species composition of the communities, as shown by the increasing proportion of cavity nesters, compared with ground nesters. Indeed, despite the lower availability of nesting resources for ground-nesters, cavity-nesters take over in high-density areas, where more concrete structures and buildings are present^15^, thus they may compensate for the loss of ground-nesting bee species.

### Wild bee community homogenization and urbanization

We did not observe any relationship between mean pairwise β-diversity and the two metrics of urbanization. This result contrasts with those of Banaszak-Cibicka and Żmihorski (2020)^44^ who found more homogeneous wild bee communities in urban environments compared to non-urban ones. Similar results have been reported for bees, with homogenization of urban pollinator communities compared to rural ones^28,45^. Biotic homogenization in urban environments has also been reported for other taxa, for example birds^46^.

**Figure 5:**
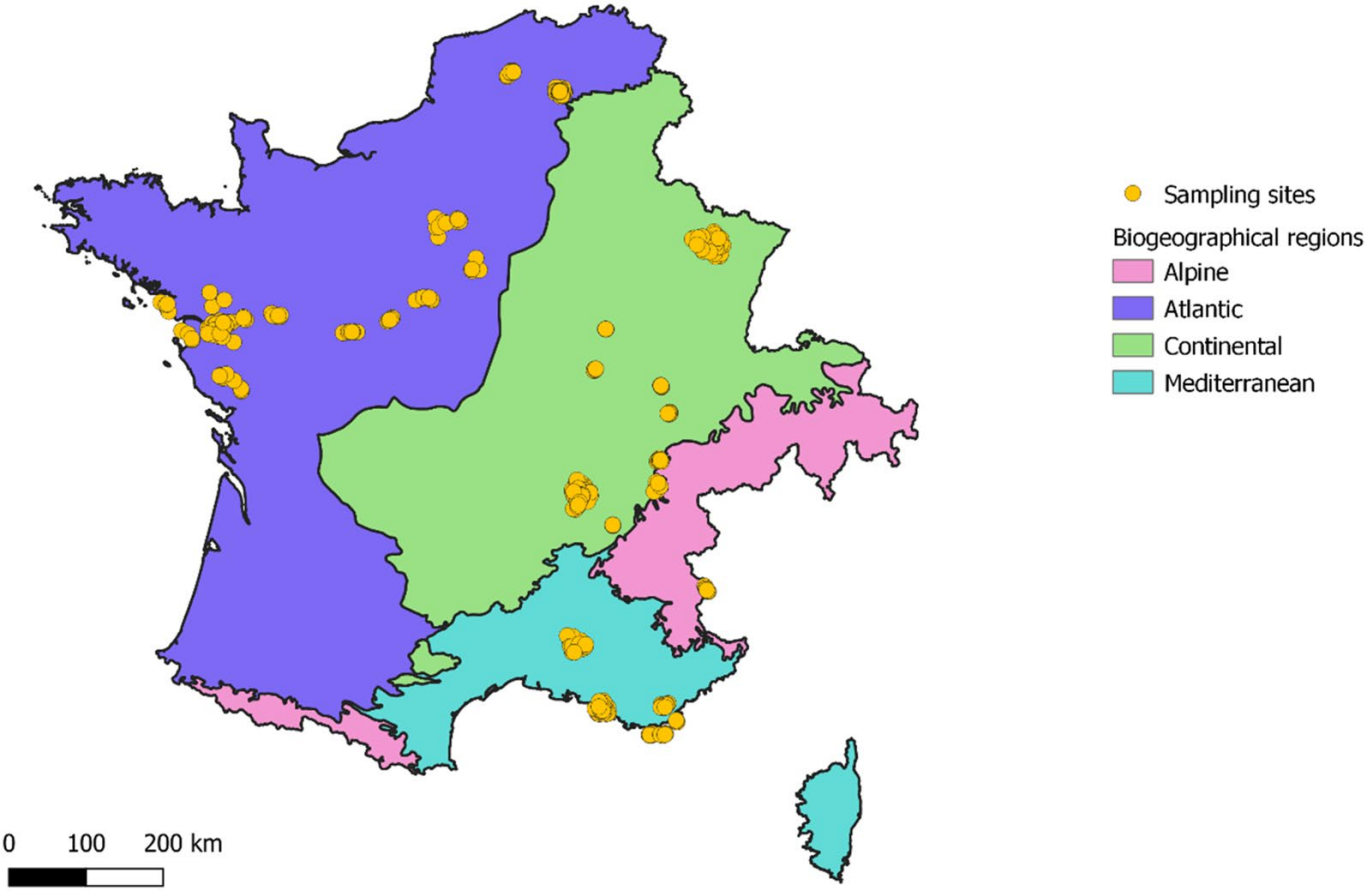
Grouped sampling sites (n = 532) in France, Belgium and Switzerland, with the biogeographical regions. In total, 238 sites belong to the Continental region, 178 to the Atlantic, 106 to de Mediterranean and 10 to the Alpine. This figure was generated using QGIS software, v3.10.13 (https://www.qgis.org/).

In our study, when considering urbanization levels, either in terms of soil sealing or human population density, urban wild bee communities are not more or less taxonomically homogeneous than non-urban ones. It is important to note that this result does not imply that urban and non-urban wild bee communities are similar, but that the homogenization of wild bee communities is constant throughout the urbanization gradient. In other words, urban communities are as dissimilar as non-urban ones. Here, the β diversity values are quite high (ranging from 0.68 to 0.96), emphasizing that even urban areas have quite dissimilar communities when compared to each other. This high level of dissimilarity among wild bee communities in urban environments can be explained by the large range of biogeographical regions encompassed in our dataset (Fig. 5), as each of these regions harbors a specific wild bee fauna^34^.

Local factors in cities might also explain these high levels of dissimilarity. We know for example that green space connectivity has effects on species richness, with more wild bee species and abundance in cities with more connected green spaces^47^. Another local explanation might come from contrasting green space management practices among cities. Not all cities have the same policies, and urban green space management is crucial to the establishment and sustainability of diverse pollinator communities^14,15,48^. Thus, we expect more dissimilar wild bee communities among cities with differing green space layout and management.

### Functional responses of bee communities to urbanization

**Figure 6:**
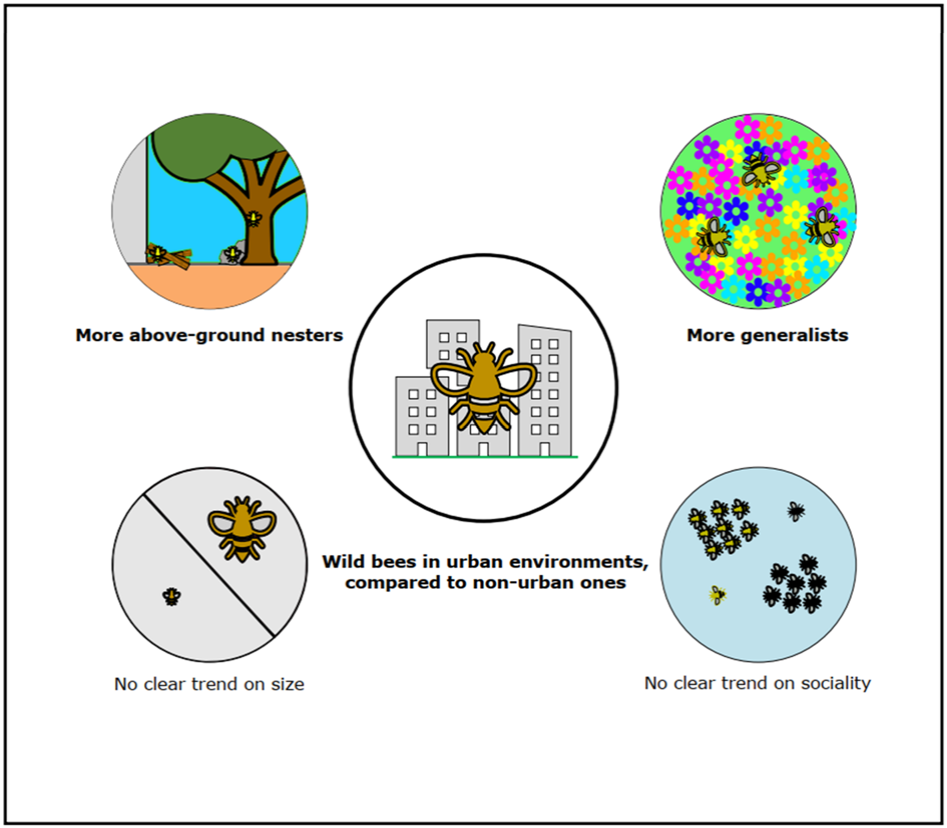
Summary picture of an urban bee community, compared to a non-urban one. This figure was generated using Inkscape v1.2 (https://inkscape.org/).

Several studies have already shown trends on how urban areas filter wild bee communities based on their functional traits (see^30^ and^49^ for reviews). However, as for taxonomic diversity, it is often difficult to identify clear variation patterns^50^. Using our large dataset, we could identify typical wild bee functional traits that are favored in urban environments, thus informing on the average functional profiles of wild bee species that may thrive in cities. We found urban wild bees in general to be typically above-ground nesters and generalists, while different trends were established for their body size and sociality, depending on the considered urbanization metric (Fig. 6).

### Nesting habits

Above-ground nesting species were more frequent with increasing urbanization than below-ground nesting ones, and this result was recorded with both urbanization metrics.

This result is consistent with what was previously reported in the literature^16,49,51,52^. Indeed, cities, with high proportions of impervious surfaces and buildings, offer fewer nesting habitats to ground-nesting species^15^, nesting sites becoming a limiting factor^39^. On the other hand, above-ground nesters can do well in cities with the presence of man-made structures, depending on their ability to use them and on their availability^53^.

The presence of green areas in cities can help ground-nesting bee species by offering more nesting opportunities and resources^17^. Several studies highlight the importance of parks and gardens in supporting bee biodiversity in cities^12,18,31,54^, which otherwise are constraining environments due to soil sealing.

### Diet

Generalist species were more frequent in more urbanized sites than specialist ones, and this was recorded for both urbanization metrics.

This is in accordance with what was previously found in the literature^32,50–52,54,55^, as specialist bee species depend on the presence of their host plants to complete their life-cycle, which are often scarce due to the rarefaction of native flowering resources. As one can find many exotic flowers in cities, especially in residential gardens and urban parks^56^, we expect to detect less oligolectic bee species in densely urbanized habitats^57^.

Notwithstanding, Banaszak-Cibicka et al. (2018)^20^ found more oligolectic species in urban parks of Poznań (Poland) compared to a national park. Thus, urban areas are not always depleted of specialist species, and well-managed parks with preserved native floral resources can obviously support specialist wild bee species in cities^58^.

Additionally, it is important to emphasize that the presence of an exotic plant species may concomitantly support an associated specialist bee species. In Poland, for instance, the spread of *Bryonia dioica* in urban environments also brought the *Andrena florea* wild bee species, specialized on this plant^59^.

### Body size

We recorded contrasting effects of the two urbanization metrics on wild bee body size: small species were more frequent in relation to higher human population density compared to large species, but we found no difference with the proportion of impervious surfaces. Contrasting impacts of urbanization on bee body size are also reported in the literature, with some studies finding little to no effect^32,50^, and some finding that urbanization often favors smaller bee species^12,30,60^. Bee body size is of particular importance because it is related to the foraging range of individuals^61,62^. In fragmented habitats, such as dense urban environments, distances between suitable nesting and feeding habitats may select for smaller species that can remain on small green spaces and rarely need to commute across several green spaces. Furthermore, small bees may be favored given that they need fewer floral resources than large bees, even though large bees can fly further^62^.

This might also explain the difference in the response of bee body size to the two urbanization metric results. In densely populated cities, it is harder to fly between suitable habitats, even for larger bees, as higher buildings and structures may act as barriers to their movement. Indeed, it has been recently shown that the 3D structure of cities impacts wild bee community composition^63^. Thus, being able to fly further might no longer be an advantage, and larger bees, requiring more floral resources than smaller ones, might be selected against. On the contrary, very impervious areas do not always host high building density (for example, as in the case of parking lots), thus making it easier for large wild bees to fly between bare soil areas.

Densely populated areas might also exhibit warmer temperatures due to the urban heat island effect, and this could, in turn, result in the selection of smaller individuals, as we know that in cities, higher temperature results in smaller body sizes^64^.

### Sociality

We also recorded contrasting effects of the two urbanization metrics on sociality: social species were more frequent in relation to higher proportion of impervious surface compared to solitary ones, but no effect was recorded with human population density. This is in agreement with a recent literature review that reports on no consensus concerning the response of this trait to urbanization^30^.

However, some urban habitats are shown to host more social species than rural habitats^20,32^, which may be linked to better reproductive success in cities compared to rural habitats such as agricultural environments^65^, an explanation that is consistent with our results on the soil sealing—sociality relationship.

### Conclusion, limits & future directions

Overall, our findings suggest that urban environment filters wild bee communities based on their functional traits. Our results also underscore different impacts of urbanization metrics on local species diversity, with a significant negative impact of soil sealing. On the contrary, both soil sealing and human population densities create strong functional filtering of trait assemblages.

These results are particularly relevant since they arise from a range of independent studies, thus providing a general view on the wild bee communities in urban environments from western Europe. Since this study covers different biogeographical zones, it further underlines its applicability to other temperate countries. We therefore expect similar patterns to shape wild bee communities in urbanized areas from other temperate regions, but further confirmatory studies would be welcome.

Our study also delivers a clear message concerning wild bee communities in urban environments. Urban environments cannot compare with non-urban ones in terms of species richness and trait diversities of bee communities. However, simple management practices of urban green spaces, such as differentiated management, or simply low management^66^, may help in maintaining this diversity. Indeed, not all green spaces are equally valuable in supporting wild bees, and pollinator assemblages in general^49^. For example, it has been shown that pollinator richness was positively influenced by green space size, but also by management measures such as mowing^67^. Increasing the quantity of floral resources and their spatio-temporal availability and diversity^40,68^ could also help conserving pollinator communities and pollination function in cities^69^, as long as these resources are native or attractive to pollinators.

We can then hypothesize that changes in managing practices could help increase functional diversity of bees in cities, with specialist and ground-nesting species being found more frequently in these low-managed urban areas.

Finally, if managing urban green space is of great importance to protect biodiversity in cities, it is crucial to involve all stakeholders, especially residents^70^ to achieve efficient and socially-accepted measures.

In the future, it will be important to consider intra-city landscape variation, and see how urban characteristics might influence taxonomic and trait diversity. This will surely allow us to better understand the dynamics shaping wild bee communities in urban environments.

## Material and methods

### Wild bee surveys and species identification

The dataset we use gathers 16 bee survey datasets obtained from a range of collaborators in France, Switzerland and Belgium. We contacted the community of bee scientists that collaborates with the *Observatoire des Abeilles*^71^, i.e., the recognized network of taxonomists from France and neighboring countries in charge of validating species identifications for the French *National Inventory of Natural Heritage*^72^. We asked bee scientists whether they would be willing to share survey datasets that met the following criteria: (i) survey datasets are recent (from 2006 onwards, with very few (10 out of 65,380 individuals) collected between 2002 and 2005), either already published and citable under the form of study reports or peer-reviewed articles, or provided as personal communications, (ii) they represent a general sampling of the bee fauna (no survey targeted a specific bee guild, such as bumblebees only, for example), (iii) each individual is identified to the species level with expert taxonomist validation, (iv) information on sampling methodology is available, and (v) surveys consist of a collection of standardized sampling sites whose coordinates are available. From all the collaborators, we gathered 16 bee survey datasets, including 838 sampling sites ranging from natural to urban areas. For each sampling site, all the individuals were identified to species level by a network of taxonomist experts and specimens were subsequently deposited in actively managed entomological collections.

Since the occurrence of the domesticated honey bee *Apis mellifera* is mostly constrained by local beekeeping practices rather than environmental variables alone, we excluded this species from the analyses.

### Species functional traits

All of the functional traits were obtained from expert knowledge (pers. comm. Roberts and Potts, 2021). We selected the following traits for our study: (i) Nesting (above *vs.* below ground), (ii) Sociality (social for eusocial and primitively eusocial *vs.* solitary), (iii) Diet (specialist for monolectic and oligolectic *vs.* generalist for polylectic species), and (iv) Size (small if ITD < 2 mm vs. large if ITD > 2 mm). The Inter-Tegular Distance (ITD) is a widely used proxy to measure bee body size, and also a good estimator of bee foraging range^62^. For each of the above traits, we added a third category “no information” whenever a species trait was unknown. This third category allowed us to include all species, even those with missing information, in our multi-trait models. The trait with the largest number of missing values was the ITD (86/469; 18%), followed by diet (36/469; 7%), nesting (31/469; 6.6%) and finally sociality (29/469; 6%).

We chose those traits as they are liable to determine species sensitivity to habitat alterations and they appear to be the most-studied in functional bee ecology^30^.

### Grouping sampling sites

We first grouped neighboring datapoints (n = 838 sampling sites) located less than 500 m apart from each other in order to reduce possible spatial dependencies, since 500 m encompasses the majority of wild bee flight distances^73^. To do so, we used the R function *hclust*, method *complete*. We computed the barycenter coordinates of grouped sites to be used as a new average site. The information on sampling methods and efforts was collated accordingly, and the new site was assigned the resulting species richness from all subsites combined. This reduced the dataset down to 532 virtually independent sampling sites (Fig. 5). All sites were successfully sampled, i.e. there was no null community sample.

### Urbanization metrics and biogeographical zones

We chose, again, a radius of 500 m to estimate urbanization metrics, for the aforementioned reasons^73^. We used two different metrics to describe urbanization around the 532 independent sampling sites.

First, we computed the *proportion of impervious surfaces*, a widely used variable to estimate urbanization^11,12,16,36^. We worked with the Corine Land Cover data repository (2012), which encompasses all of Europe and is thus consistent throughout the different sites. As “impervious surfaces”, we considered the following Corine Land Cover categories: continuous and discontinuous urban fabric, industrial or commercial units and public facilities, road and rail networks and associated lands, airports, mineral extraction sites, dump sites, construction sites, and sports and leisure facilities.

Secondly, we computed the *human population density* as an alternative urbanization metric. To calculate this variable around the sampling sites, we used the geographic information from the human population density raster (100 m resolution) computed by Gallego, 2010^74^. The human population density geographic layer covers Europe as a whole, and is therefore equally available for sites in France and Belgium. Concerning the eight sites in Switzerland, we used the corresponding raster data from GEOSTAT, 2018^75^ to get the human population density in Geneva.

Moreover, we assigned each site to a biogeographical region because bee diversity is obviously influenced by biogeography^34^. Our study zone encompasses four biogeographical regions: Continental, Mediterranean, Atlantic and Alpine (Fig. 5).

### Statistical analyses

#### Controlling for sampling heterogeneity

One critical challenge for synthesis research, such as in the present study, is to control for heterogeneity of sampling efforts and methods across individual surveys. Some sampling sites may also combine two or more sampling methods.

As achieving consistent sampling efforts throughout sampling sites is not feasible, we favored a fuzzy-coding approach whereby sites were classified into broader categories of sampling schemes and accounting jointly for sampling methodology, effort and temporal span. To do so, we used a set of three methodological variables: the number of days of active sampling per site (capture by either net, box or a few direct observations), total duration of passive sampling per site (passive sampling including pan traps and malaise traps) and if the site had been sampled with kick nets (0/1, binary variable).

We then summarized this sampling information by clustering the sampling sites according to the three aforementioned methodological variables, in order to assign sites into broad sampling scheme categories. To achieve the most parsimonious clustering, we compared results of two widely used clustering methods: k-means clustering and hierarchical clustering, using the R function *NbClust* (*NbClust* package, v3.0^76^). We found three optimal clusters for k-means and four for hierarchical clustering. The k-means method clustered the sites primarily on the basis of passive sampling effort, while differences in active sampling effort were most evident in hierarchical clustering. This resulted in somewhat different clusters for the two methods, with clusters differing in their sampling methods and sampling effort. Thus, we combined both clustering methods, and achieved a final number of five sampling categories, each site being assigned to one sampling category (Fig. A1). The sampling categories were taken to be broad sampling scheme categories, allowing for statistical control of sampling heterogeneity among survey datasets in subsequent analyses.

#### Dealing with imperfect species detection

Sampling species-rich communities like bees is arguably subject to imperfect species detection, i.e. false absence data leading to imperfect species sampling coverage. This may somehow affect the accuracy of species occurrence models and their ability to detect spatial patterns of diversity and functional trait occurrence. We followed three general precautions to limit this problem in diversity and functional trait analyses. First, we exclusively used species presence/absence data instead of abundance, to reduce biases arising from sampling effort variations. Second, whenever relevant, we accounted for the different sampling scheme categories as a random variable in statistical models. Indeed, sampling sites from different sampling categories may on average achieve different sampling coverage, which should be controlled for. Third, to ensure that actual species coverage levels provided an overall satisfactory overview of the diversity of bee communities, we computed species richness accumulation curves for each of the 532 sampling sites (R package iNEXT, v2.0.20^77^). We found that samples covered an average of 62% of expected local species richness values, which we found reasonably high to perform meaningful analyses of diversity and functional trait spatial patterns.

#### Variations in community species richness with urbanization (α diversity)

Species richness in a site is defined as the number of species inventoried in that site. To analyze this variable in relation to urbanization, we used a mixed effects model with spatial random effect, from the *spaMM* package, v3.12.0^78^ because we detected strong spatial autocorrelation in the residuals of the non-spatial models (*moran.test*, from *spdep* package, v1.1.11^79^). As we have overdispersed count data, with an absence of zero, we used a truncated negative binomial distribution for this model.

We considered both the human population density and proportion of impervious surfaces in the same model for community species richness analysis, plus the biogeographical region, and the sampling-scheme category as a random variable. We first checked that the urban population density and the proportion of impervious surfaces were not correlated using the Variance Inflation Factor (R function *vif* from the package *car*, v3.0.11^80^; VIF = 1.57, r = 0.62). The VIF quantifies the variance increase caused by correlation between variables. Typically, a value below 5 between variables is not considered to be a problem. Furthermore, as shown in Fig. A1, habitats with the highest proportions of impervious surfaces encompass a wide range of human population densities: from 110 to more than 25 000 inhabitants per km^2^.

#### Variation of community taxonomic diversity with urbanization (β-diversity)

β-diversity measures (dis)-similarity between communities. The objective of this analysis is to compare mean pairwise β-diversity among sites along an urbanization gradient, to test whether heavily-urbanized sites have more homogenous communities than less-urbanized ones. To do so, we used the Sorensen dissimilarity index (R function *betadiver*, index “w”, package *vegan*, v2.5.7^81^) for presence/absence data, and computed two different models depending on the urbanization metric: (i) linking β-diversity to the human population density and (ii) linking β-diversity to the proportion of impervious surfaces.

Concerning the human population density, we applied a log(x + 1) transformation so as to reduce orders of magnitude and to achieve a more tractable scaling of the values. The resulting human population density scale ranged from 0 to 10.3 units. We then categorized our sites based on their human population density by splitting this variable into intervals of size 0.2, which was the finest resolution our dataset could afford given site numbers (for a total of 53 intervals, with an average of 10.03 ± 5.55 sites per level, a minimum of 0 and a maximum of 27). This splitting allowed us to evaluate the relationship between wild bee communities’ homogeneity and urbanization. The mean within-interval β-diversity values were computed within urbanization levels, with the underlying hypothesis that taxonomic homogenization would result in ever-decreasing β-diversity as urbanization levels increased. This homogenization hypothesis was tested using a linear model (lm) and assuming a Gaussian error distribution for this model given that β-diversity residuals were normally distributed (Shapiro test, *p* = 0.78). We ensured beforehand that no confounding effect would arise from the number of sites per urbanization level, from the mean Euclidean geographical distance among those sites, by checking for possible collinearity with urbanization levels. To do so, we performed correlation tests (*cor.test*), and none of them were found significant (see Supplementary A4). Levels comprising less than two sites could not be considered in this β-diversity analysis.

The same analytical scheme was used to investigate possible links with β-diversity and the second urbanization metric, namely the proportion of impervious surfaces, which varies from 0 to 100%. We again split this variable into intervals of size 2% (for a total of 51 intervals, with an average of 10.4 ± 27.5 sites per level, minimum of 1 and maximum of 188). For this second model, we also assumed a Gaussian error distribution (Shapiro test, *p* = 0.93). Again, correlation tests were performed between the proportion of impervious surface intervals and the mean pairwise Euclidean geographical distance and the number of sites considered in the analysis. This time, the correlation was significant between the proportion of impervious surfaces and the mean pairwise Euclidean distance (*p* = 0.0016, Supplementary A4). However, since the coefficient of correlation remained weak (r = − 0.43), we assumed no relation between these two variables.

#### Variation of functional trait occurrences with urbanization

In addition to the taxonomic homogenization hypothesis, we investigated the ecological filtering hypothesis, whereby some bee functional traits may be favored by urbanization. We expected that functional traits of bee species reported from highly urbanized sampling sites would not be a random subset of the global species pool covered by our study. Following Fortel et al. (2014)^16^, each species of the global species pool was assigned a presence-absence binary occurrence record for each sampling site. The resulting species occurrence probability values were modeled as a function of interacting terms of their functional traits with urbanization metrics, using a binomial generalized linear mixed model (glmm) framework. We built two different models, whose explanatory variables included functional traits (nesting, size, sociality and diet), urbanization metrics differing between the two models (either human population density or proportion of impervious surfaces), their two-way interactions, as well as biogeographical categories to account for possible variability arising from regional effects. A significant interaction between an urbanization metric and a given functional trait would reveal that the occurrence of species along the urbanization gradient is not randomly drawn from the global species pool, but is rather partly driven by their functional characteristics.

Additional factors were implemented as random grouping variables to account for random statistical noise or non-independence of occurrence records. First, sampling sites, nested within their corresponding methodological sampling category, were used to specify an appropriate grouping structure of occurrence data. Second, as species may be viewed as separate strata in the occurrence analysis, their identity was also specified as an additional random grouping variable.

In these trait analyses, we did not consider parasitic species, since their presence depends on their host and not solely on environmental variables, which would require further analyses. Furthermore, the specimens of this peculiar functional group have not been systematically assigned a species in the original datasets, precluding any analysis of their occurrence patterns.

In this section, the binomial glmm analysis was performed using the *glmmTMB* function from the *glmmTMB* package, v1.1.2^82^, and *p* values were adjusted using the *False Discovery Rate*^83^ methods.

All general linear model residuals were evaluated using the *DHARMa* package, v0.4.3^84^.

All statistical and GIS analyses were carried out in R v4.1.1^85^.

## Supplementary Information

Supplementary Information 1.

Supplementary Information 2.

## Data Availability

All data and R codes are available upon request to the corresponding author.
