# Supplementary material for: A large-scale dataset reveals taxonomic and functional specificities of wild bee communities in urban habitats of Western Europe
Source: Sci Rep. 2022 Nov 7;12:18866. doi: 10.1038/s41598-022-21512-w (PMC9640672; doi:10.1038/s41598-022-21512-w)
Supplement: Supplementary file 1 — Supplementary Information 1. [file 41598_2022_21512_MOESM1_ESM.docx]

**Supplementary A1.** Relation between proportion of impervious surfaces and human population density.


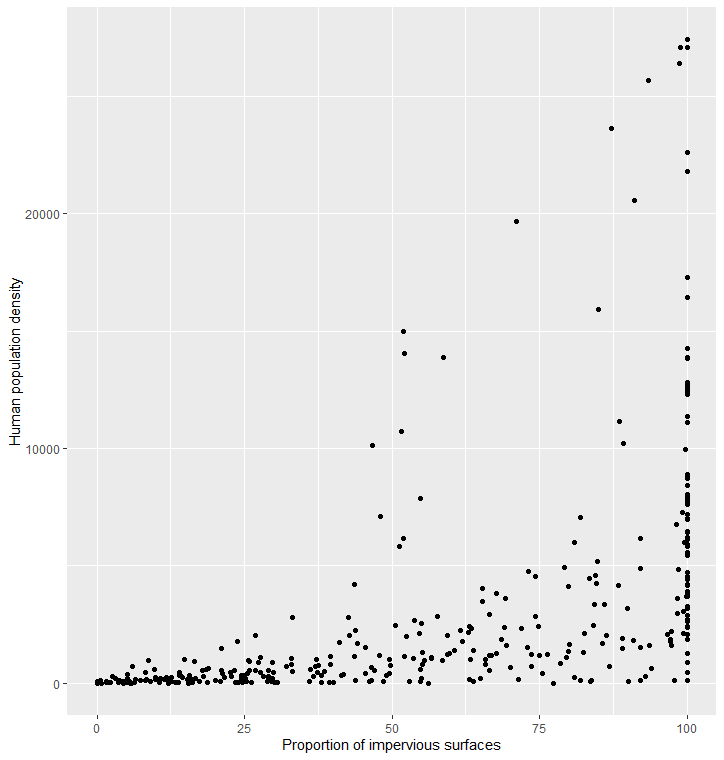


**Figure A1.** Human population density – Proportion of impervious surfaces (both in a 500m radius) relation across the 532 clustered sampling sites.

**Supplementary A2.** Sampling extensiveness clusters

**Figure A2.** Plot of passive sampling extensiveness (total number of hours of passive sampling) vs active sampling extensiveness (total number of days of active sampling), and clusters achieved using the k-means and the hierarchical clustering methods. The binary variable accounting for the presence/absence of kick nets is not represented herein, but accounts for the splitting of cluster 1 vs. cluster 5. Red dots: cluster 1; Brown dots: cluster 2; Green dots: cluster 3; Blue dots: cluster 4; Purple dots: cluster 5


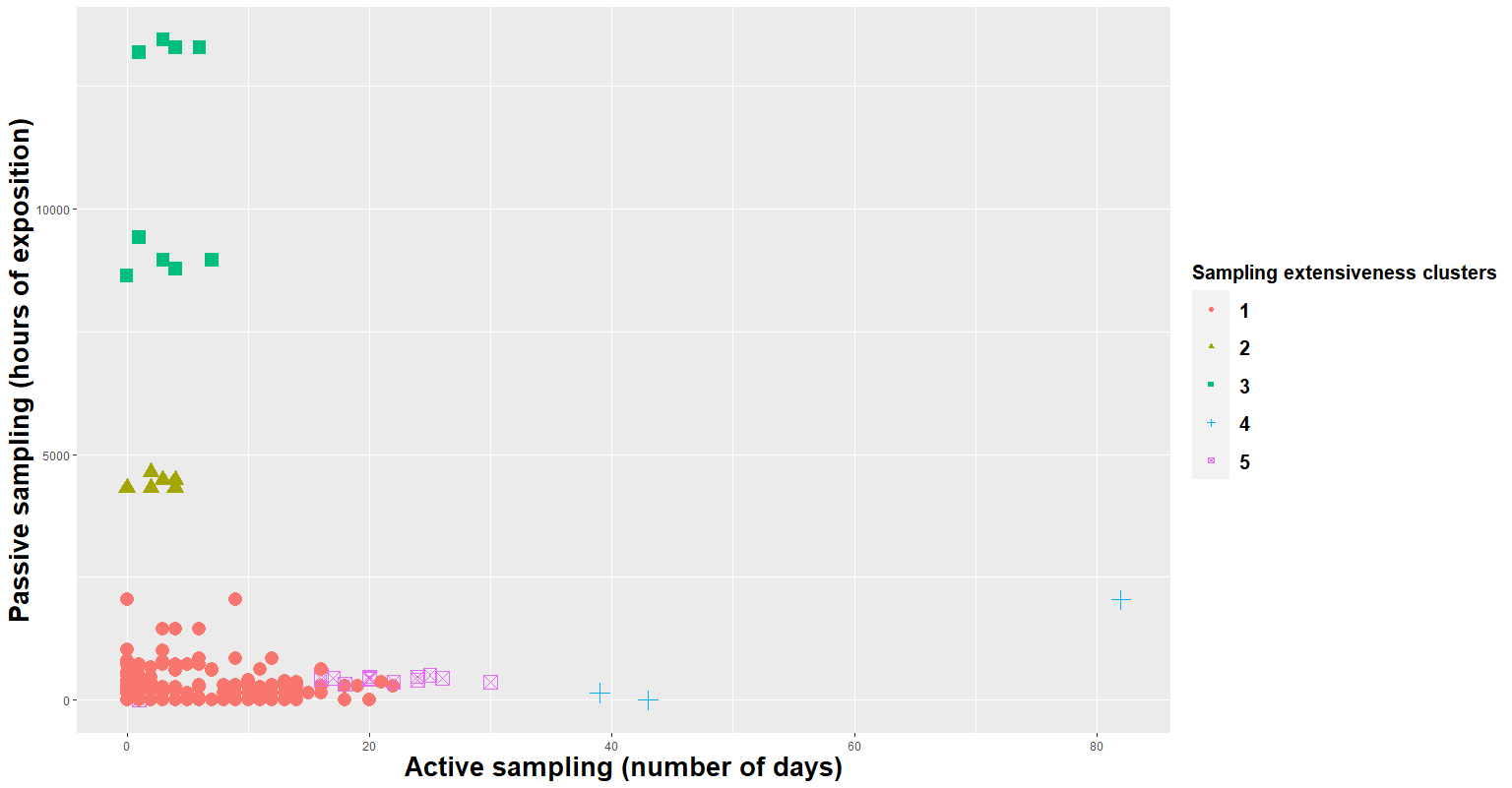


**Supplementary A3.** Model equations

- **Species richness model** (spaMM, 0-truncated negative binomial distribution)

***Species richness = % impervious surfaces + Human population density + Biogeographical zone + (1|Sampling_clusters)***

- **Beta diversity models** (lm, gaussian distribution)

***Mean beta diversity = Human population density***

***Mean beta diversity = % impervious surface***

- **Trait models** (glmmTMB, binomial distribution)

***Species presence/absence = Human population density * (nesting + diet + size + sociality) + Biogeographical zone + (1|species) + (1|sampling clusters/cluster ID)***

***Species presence/absence = % impervious surfaces* (nesting + diet + size + sociality) + Biogeographical zone + (1|species) + (1|sampling clusters/cluster ID)***

**Supplementary A4.** Correlation tests results for the beta diversity models

| **Correlation tested** | **Correlation coefficient** | **t-value** | **p-value** |
| --- | --- | --- | --- |
| *Mean Euclidean distance*Human population density* | -0.26 | -1.89 | 0.063 |
| *Number of sites*Human population density* | -0.019 | -0.14 | 0.89 |
| *Mean Euclidean distance*% impervious surfaces* | -0.43 | -3.33 | **0.002** |
| *Number of sites*% impervious surfaces* | -0.15 | -1.09 | 0.28 |

**Table A1**. Results of the Pearson’s correlation tests between urbanization metrics and the mean Euclidean distance between sites or the number of sites falling in the considered urbanization category. The p-value in bold are significant (alpha = 5%).
